# Dynamic Characterisation of Fibre-Optic Temperature Sensors for Physiological Monitoring

**DOI:** 10.3390/s21010221

**Published:** 2020-12-31

**Authors:** Joanna M. Coote, Ryo Torii, Adrien E. Desjardins

**Affiliations:** 1 Department of Medical Physics and Biomedical Engineering, University College London, Gower Street, London WC1E 6BT, UK; a.desjardins@ucl.ac.uk; 2 Wellcome/EPSRC Centre for Interventional and Surgical Sciences, University College London, Charles Bell House, 43–45 Foley Street, London W1W 7TY, UK; 3 Department of Mechanical Engineering, University College London, Torrington Place, London WC1E 7JE, UK; r.torii@ucl.ac.uk

**Keywords:** temperature sensors, fibre-optic sensors, dynamic response, time constant, minimally invasive surgery, physiological monitoring, interventional devices

## Abstract

Fast, miniature temperature sensors are required for various biomedical applications. Fibre-optics are particularly suited to minimally invasive procedures, and many types of fibre-optic temperature sensors have been demonstrated. In applications where rapidly varying temperatures are present, a fast and well-known response time is important; however, in many cases, the dynamic behaviour of the sensor is not well-known. In this article, we investigate the dynamic response of a polymer-based interferometric temperature sensor, using both an experimental technique employing optical heating with a pulsed laser, and a computational heat transfer model based on the finite element method. Our results show that the sensor has a time constant on the order of milliseconds and a −6 dB bandwidth of up to 178 Hz, indicating its suitability for applications such as flow measurement by thermal techniques, photothermal spectroscopy, and monitoring of thermal treatments.

## 1. Introduction

Many applications require fast miniature temperature sensors. Biomedical examples include intravascular flow measurement by thermodilution [1,2,3,4], where cold saline is injected into the artery and the flow rate is determined from temperature changes measured by a downstream sensor; and hot-wire anemometry, in which the temperature of a heated element depends on the rate of flow of the gas [5,6,7,8,9,10] or liquid [11,12,13,14] surrounding it. In therapies such as laser or radiofrequency ablation [15,16,17,18,19,20,21,22,23,24], temperature monitoring is critical to ensure complete ablation of the diseased tissue while leaving healthy tissue undamaged; in photothermal spectroscopy [25,26,27,28,29,30,31], chemical species are identified by the temperature variations caused by optical absorption of a modulated light source. These applications require measurement of rapidly varying temperatures and, therefore, temperature sensors with both fast and well-known response times are required.

Many of the aforementioned techniques are performed as minimally invasive interventional procedures, in which highly miniaturised and flexible sensors are needed for integration into catheters, needles and guidewires. Fibre-optics can readily meet these requirements, and fibre-optic temperature sensing approaches include fibre Bragg gratings (FBG) and long period fibre gratings (LPFG) [32,33,34,35,36,37]; polymer-based [38,39,40,41,42,43,44,45,46,47], inorganic [48,49,50] and microbubble-based [51] Fabry–Pérot (FP) cavities; multimode interference (MMI) segments [52,53,54,55,56,57,58]; infiltrated photonic crystal fibre and hollow-core fibre [59,60]; fluorescence-based methods [22,23,24]; and sensors based on polymer optical fibres [61,62,63,64]. The wide variety of geometries and materials employed in these sensors can lead to very different response times, from sub-millisecond for a silicon FP cavity [50] to hundreds of milliseconds for packaged FBGs [18,36].

We have developed FP-based interferometric sensors with optical cavities made from a thermally sensitive polymer, for monitoring of coronary blood flow using a thermal tagging technique [2]. The high thermal expansion coefficient of the polymer material (340 ppm/K [65], compared to 2.6 ppm/K for silicon [66] and 0.41 ppm/K for fused silica [67]) permits high temperature sensitivity while maintaining small sensor element dimensions (axial and radial dimensions on the order of tens of microns).

Although polymer FP cavity-based fibre-optic temperature sensors have been investigated [38,39,40,41,42,43,44,45,46,47], many of these studies have not considered the response of the sensors to rapid, dynamic temperature changes. Characterising the dynamic response can be challenging: a common method is to rapidly immerse the sensor into water at a different temperature to the ambient room temperature, providing temperature step input [17,18,35,36,50]. However, limitations to this technique include errors caused by unknown temperature gradients in the air and water, uncontrolled speed and depth of immersion [68], and temperature gradients in the fibre that would not be present in normal use. The speed of response is also strongly dependent on the thermal properties of the medium surrounding the sensor, and it is challenging to study this dependence using the immersion method. Furthermore, in polymer-based sensors, water absorption into the polymer elements can be significant [32,62,69] and therefore a sudden change of surrounding medium (e.g., air to water, or vice versa) could add an unwanted contribution to the sensor response. A ramped temperature input can also be used for measuring the dynamic temperature response [37,68,70]; however, the minimum measurable time constant is limited by the gradient of the ramp [68]. Other approaches include heat-transfer modelling [35], and optical heating methods: several investigators have studied the dynamic responses of thermocouples and resistance thermometers using pulsed or modulated light to induce rapid temperature cycles [71,72,73,74,75].

Here, we present an investigation of the dynamic response of our fibre-optic temperature sensor, using an experimental method based on optical heating. We compare these results with a computational thermal model based on the finite-element (FE) method. Both methods show that the sensors have response times on the order of milliseconds, indicating their suitability for coronary flow measurement and other applications where response speed is critical. This article is arranged as follows: Section 2 describes the sensor construction and mechanism, and provides a simple theoretical analysis of the static and dynamic sensor responses. Section 3 presents static calibration of the sensors; Section 4 presents the experimental characterisation of the sensors’ dynamic response; Section 5 presents the FE thermal model and compares the simulated and experimental results; and Section 6 provides a discussion and summary of the work. All raw data presented in this study are available online (see Supplementary Materials).

## 2. Sensor Description

**Figure 1:**
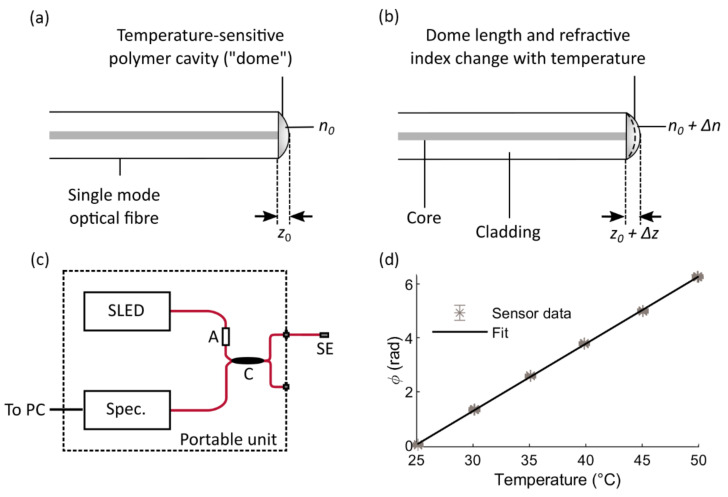
(**a**) Sensor schematic; (**b**) illustration of sensor operating principle. In both sub-figures, *z*_0_ is the initial length of the dome; Δ*z*: a small temperature-induced change in dome length; *n*_0_: the initial refractive index of the dome material; Δ*n*: a small temperature-induced change in the dome refractive index. (**c**) Sensor interrogation setup; SLED: superluminescent light emitting diode; A: attenuator; C: 50:50 fibre-optic coupler; Spec.: spectrometer; SE: sensor element; red lines indicate single-mode fibre-optic cables. (**d**) Temperature calibration plot, where *ϕ* represents the phase (complex argument) of the interference pattern received by the spectrometer [45]; the solid black line indicates the least-squares line of best fit and the error bars indicate the standard deviation of *ϕ* at each temperature step.

Sensors were constructed from single-mode optical fibre (SM800, Thorlabs, Newton, NJ, USA), cleaved at 90° to the optical axis. A droplet of polydimethylsiloxane (PDMS, Sylgard 184, Dow, Midland, MI, USA) was deposited on to the cleaved facet, and formed a hemisphere shape, hereafter referred to as a “dome”. Once cured, the dome formed a low-finesse optical cavity whose optical path length changed in response to temperature, via thermal expansion and the thermo-optic effect. Figure 1a shows a diagram of the sensor construction and Figure 1b illustrates the sensor operating principle.

At the beginning of a measurement, the length of the dome is denoted as $z_{0}$ (measured along the optical axis); thermal expansion causes a small change, Δz, in this length. Similarly, $n_{0}$ is the refractive index of the dome material at the interrogation wavelength, at the beginning of the measurement, and a change in temperature causes a small change in the refractive index, Δn, due to the thermo-optic effect. These quantities are illustrated in Figure 1a,b. Temperature changes therefore induce changes in the optical path length of the dome, which may be expressed as $\left(n_{0}\;\;+\;\;\Delta n\right)\left(z_{0}\;+\;\Delta z\right)$.

The optical path length of the dome is monitored with high resolution using phase-resolved low coherence interferometry (LCI) [76], using the setup shown schematically in Figure 1c. The interrogation system and signal processing methods have been described in more detail in a previous publication [45]. Briefly, light from a broadband superluminescent light-emitting diode (SLED, BLM-S-820-B-I-10, Superlum, Carrigtwohill, Ireland, central wavelength 830 nm), propagates in the sensor fibre and is reflected at two interfaces: the first between the fibre end-face and the dome, and the second between the dome and the surrounding medium. Reflected light from these interfaces travels back to a compact optical spectrometer via a 50:50 fibre-optic coupler (TW850R5A2, Thorlabs, Newton, NJ, USA), and the optical path difference between the reflected waves results in interference. This optical path difference may be expressed as $z^{\prime }\approx 2n_{0}z_{0}\;+\;2\left(\Delta nz_{0}\;+\;n_{0}\Delta z\right)=z_{0}^{\prime }\;+\;\Delta z^{\prime }$, or twice the optical path length of the dome, and the approximation is valid provided $\Delta n\;<<\;n_{0}$ and $\Delta z\;<<\;z_{0}$.

The intensity spectrum detected by the spectrometer (Flame-S, Ocean Optics, Orlando, FL, USA) consists of the spectrum of the SLED, modulated by a pattern of interference fringes. The initial optical path difference, ${z^{\prime }}_{0}$, can be found by converting the spectrum from the inverse wavelength (k) domain to the optical distance ($z^{\prime }$) domain using an inverse Fourier transform (IFT), and finding the optical distance at which the maximum complex magnitude of the IFT occurs. The complex argument of the IFT at this maximum is related to the small changes in the optical path length caused by temperature changes ($\Delta z^{\prime }$) as follows [45,76]:

(1) $$\phi \left(t\;-\;t_{0}\right)=\arg\left\{{ℑ}^{\;-\;1}\left[I\left(k\right)\right]\left(\;+\;z_{0}^{\prime }\right)\right\}=\frac{2\pi }{\lambda_{c}}\Delta z^{\prime }\left(t\;-\;t_{0}\right)$$

where ${ℑ}^{-1}$ indicates the inverse Fourier transform, $\lambda_{c}$ is the central wavelength of the SLED spectrum, and the complex argument ϕ is measured as a function of time t, relative to an arbitrary start time $t_{0}$. Equation (1) shows that the complex argument $\phi \left(t\;-\;t_{0}\right)$ is proportional to $\Delta z^{\prime }$. In practice, the complex argument is taken as the sensor signal, and is calibrated to temperature without performing the conversion to $\Delta z^{\prime }\left(t-t_{0}\right)$. Note that $\phi \left(t\;-\;t_{0}\right)$ is a relative measurement; to obtain absolute temperature measurements, the initial phase offset $\phi \left(t_{0}\right)$ must be measured along with a reference temperature measurement taken at $t=t_{0}$. For brevity, from this point onward we will use ϕ to signify $\phi \left(t-t_{0}\right)$.

### 2.1. Theoretical Analysis of the Sensor Response

#### 2.1.1. Static Sensitivity

The sensitivity of the sensor signal to temperature may be expressed as:

(2) $$\frac{d\phi }{dT}=\frac{2\pi }{\lambda_{c}}\frac{d}{dT}\left(\Delta z^{\prime }\right)=\frac{4\pi }{\lambda_{c}}\frac{d}{dT}\left(\Delta n\left(T\right)z_{0}\;+\;n_{0}\Delta z\left(T\right)\right)$$

In Equation (2), $d/dT\;\left(\Delta z\left(T\right)\right)$ is given by:

(3) $$\frac{d}{dT}\left(\Delta z\right)\approx \frac{\Delta z}{\Delta T}\approx z_{0}\alpha$$

where α is the linear thermal expansion coefficient of the dome material. Here we assume that $\Delta z\;<<\;z_{0}$. $d/dT\;\left(\Delta n\left(T\right)\right)$ is the thermo-optic coefficient of the dome material. The static temperature sensitivity of the sensor signal, denoted $S_{T}$, may therefore be written:

(4) $$S_{T}=\;\frac{d\phi }{dT}=\frac{4\pi }{\lambda_{c}}\left(z_{0}\frac{dn}{dT}\;+\;n_{0}z_{0}\alpha \right)$$

Therefore, we expect the sensor signal to be linear with temperature, assuming that the thermo-optic coefficient and thermal expansion coefficient are constant over the temperature range of interest.

#### 2.1.2. Dynamic Behaviour

Here, we assume that that the main contributor to the sensor’s response speed is the time taken for heat to transfer from the surrounding medium to the dome. We further assume that the temperatures of the dome and of the medium surrounding the sensor are homogeneous and isotropic, but varying in time, and that the volume, surface area, density, specific heat capacity of the dome, and the heat transfer coefficient between the dome and surrounding medium are constant and independent of temperature. Moreover, we assume that heat transfer takes place only from the surrounding medium to the dome, i.e., there is no heat loss from the dome.

Based on the conservation of energy, the heat flowing into the dome during a small time interval dt must be equal to the energy stored in the dome. This may be expressed as:

(5) $$UA\left(T_{m}\;-\;T_{d}\right)dt=V\rho CdT_{d}$$

where U is the heat transfer coefficient between the surrounding medium and dome in Wm^−2^K^−1^; A is the surface area across which heat transfer takes place; $T_{m}$ is the temperature of the medium; $T_{d}$ is the temperature of the dome; V is the volume of the dome; ρ is the density of the dome, and C is the specific heat capacity of the dome. Using Equation (4), we obtain:

(6) $$\frac{V\rho C}{UA}\frac{d\phi }{S_{T}}=\left(T_{m}\;-\;T_{d}\right)dt$$

and

(7) $$\tau \frac{d\phi }{dt}\;+\;\phi =S_{T}T_{m}$$

where τ = VρC/UA is the time constant of the sensor. This analysis models the sensor as a first-order instrument, where under static temperature conditions, the sensor response depends only on the static sensitivity $S_{T}$, and under dynamic conditions, the speed of the sensor response depends entirely on the time constant τ.

The dynamic responses of first-order instruments are well-known [77], and can be derived by solving Equation (7), with the appropriate initial conditions and time-dependent function substituted for $T_{m}$. The standard inputs considered here are a temperature step change, a temperature impulse and a sinusoidal temperature variation.

For a step change, we assume the initial condition that ϕ=0 at t=0. At t=0, the temperature of the medium instantaneously increases by an amount $T_{step}$. Setting $T_{m}$ equal to $T_{step}$ in Equation (7), and using the initial condition, we obtain:

(8) $$\phi =S_{T}T_{step}\left(1\;-\;\exp\left(\;-\;t/\tau \right)\right)$$

For a temperature impulse, the sensor is subjected to a temperature profile of the form: $T_{m}\left(t\right)\;=\;R\delta \left(t\right)$, where $\delta \left(t\right)$ is the Delta function and R is a scale factor representing the strength of the impulse. Again, the initial condition is that ϕ=0 at t=0, and the impulse response function is:

(9) $$\phi =\frac{S_{T}R}{\tau }\exp\left(\;-\;t/\tau \right)$$

Finally, the steady-state response of the sensor to a sinusoidal temperature input of the form $T_{m}=T_{0}sin\left(\omega t\right)$, where $T_{0}$ is the amplitude of the sinusoidal temperature input and ω is the angular frequency, is given by [78]:

(10) $$\phi =\frac{S_{T}T_{0}}{\sqrt{1\;+\;\omega^{2}\tau^{2}}}\sin\left(\omega t\;+\;\theta \right)$$

where θ is the phase lag of the sensor response, given by $\tan^{\;-\;1}\left(-\;\omega t\right)$, and the ratio of the amplitude of the sensor response to the input is given by $S_{T}/\sqrt{1\;+\;\omega^{2}\tau^{2}}$.

## 3. Static Characterisation

Sensors were placed in a temperature calibrator (TCL-3M165E, Omega Engineering, Manchester, UK), containing a stirred temperature-controlled water bath with a nominal uncertainty of ±0.1 °C. The water bath temperature was set to six temperatures, in the following order: 45 °C, 50 °C, 40 °C, 25 °C, 35 °C, and 30 °C, so that the sensors were subjected to repeated heating and cooling cycles during the experiment. Each temperature was maintained for one minute after stabilisation. Signals from the sensor under test were recorded in real-time, at a mean sampling rate of 76 Hz, using a custom signal and acquisition programme written in LabVIEW 2017 (National Instruments, Austin, TX, USA).

The data were processed using Matlab R2020a (MathWorks, Natick, MA, USA). At each temperature step, 4000 samples were averaged, representing a time period of approximately 53 s. The averaged sensor signals were plotted against the water bath temperatures as shown in Figure 1d, and the sensitivity, in rad/°C, was obtained from the gradient of the line of best fit. In the case of the sensor shown in Figure 1d, the sensitivity is 0.248 ± 0.008 rad/°C, where the uncertainty represents the 95% confidence interval in the slope of the linear fit. Two other sensors were calibrated in the same way, and were found to have sensitivities of 0.222 ± 0.004 rad/°C and 0.238 ± 0.002 rad/°C. All R^2^ values were greater than 0.99. In all of the sensors tested, no evidence of hysteresis or drift was observed during the temperature cycling.

The sensor readings can be converted into a temperature change using the relation $T\left(t\;-\;t_{0}\right)=\;\phi /S_{T}$. The uncertainties in the temperature measurements were estimated by propagation of the uncertainties in the measured sensor signals and the sensitivities, and were estimated as 2%, 5% and 2% of reading, respectively, using 95% confidence intervals.

The detection limit for each sensor was calculated according to Method I in reference [79]: the minimum detectable temperature change was defined as a temperature change equal to the uncertainty in the measured temperature, which was found by dividing the standard deviation of the 4000 measurements taken at 35 °C by the sensitivity obtained from the calibration curve, and multiplying by a factor of two to allow for 95% confidence intervals. The detection limits were found to be 0.05 °C, 0.1 °C and 0.08 °C, respectively.

## 4. Dynamic Characterisation: Experiment

### 4.1. Experimental Setup

For the experimental determination of the time constant, new dome sensors were made, using double clad fibre (DCF 13, Thorlabs, Newton, NJ, USA). DCF has a thin core that acts as a single-mode waveguide, a first cladding layer that acts as a multimode waveguide, and a second cladding layer that provides optical confinement of the guided modes. The core was used for delivery of the interrogation light, and the first cladding was used to deliver pulsed laser light at a wavelength of 532 nm to the dome.

A pigment (Silc Pig™, Smooth-On, Macungie, PA, USA) was added to the polymer forming the dome, by hand-mixing the pigment with the uncured liquid PDMS. Prior to mixing, the pigment and PDMS were weighed out in ratios of between 0.1 % and 0.9 % pigment to PDMS, using a high-precision balance (SI-234, Denver Instrument, Bohemia, NY, USA), and after mixing, the mixture was degassed in a vacuum chamber (5305–0910, Thermo Scientific, Waltham, MA, USA) to remove bubbles introduced by mixing.

The chosen pigment absorbed light strongly at the wavelength of the pulsed laser (532 nm), but was much less absorbing at the wavelengths emitted by the interrogation source (in this case 1550 nm). Therefore, the pulsed laser light was absorbed by the pigment, resulting in sudden and short-term heating of the dome over the duration of the laser pulses.

The absorption spectrum of the pigment-containing polymer at different concentrations was measured with a spectrophotometer (LAMBDA™ 750 UV/Vis/NIR (ultraviolet/visible/near-infrared), PerkinElmer, Waltham, MA, USA), and the absorption coefficient at 532 nm, μ, was found to have a linear relationship to percentage concentration of pigment by weight, *c*, of the form μ = 13c  +  0.078 mm^−1^.

It was assumed that before the laser pulse, the dome, fibre and medium were in thermal equilibrium. Because the laser pulse duration was much smaller than the expected time constant, the temperature change it produced in the dome could be approximated as an impulse. Therefore, we expected the sensor to exhibit the first-order instrument impulse response described by Equation (9), and the time constant τ could be obtained by fitting a curve of this form to the sensor data.

**Figure 2:**
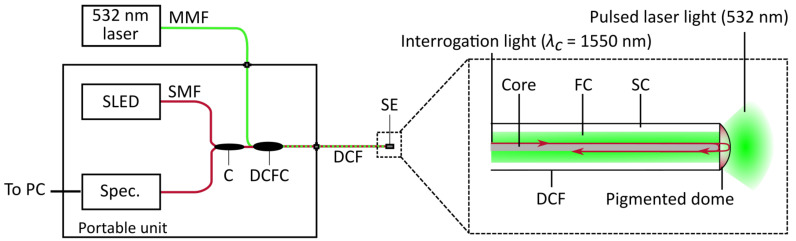
Diagram of the setup for the optical heating experiment. Left: schematic of the combined interrogation and pulsed laser excitation system; MMF: multimode fibre; SLED: superluminescent light-emitting diode; SMF: single mode fibre; Spec.: spectrometer; C: circulator; DCFC: double-clad fibre coupler; DCF: double-clad fibre; SE: sensor element. Right: enlarged schematic of double-clad fibre sensor element; FC: first cladding; SC: second cladding.

The sensors were connected to an LCI interrogation setup as described in Section 2, and to the pulsed laser (FQ-200-20-V-532, Elforlight, Daventry, UK), via a double-clad fibre coupler (SFO5132-TFB-550212B33-001, Gooch and Housego, Ilminster, UK). In this experiment, the interrogation light source was an SLED with a central wavelength of 1550 nm (EBD260089-03, Exalos, Zürich, Switzerland), the 50:50 fibre-optic coupler was replaced by a circulator (6015-3-APC, Thorlabs, Newton, NJ, USA), and a spectrometer with a wavelength range of 1510–1595 nm was used (I-MON USB, Ibsen, Farum, Denmark). The laser was pulsed at a repetition rate of 25 Hz, with a pulse duration of 10 ns. Figure 2 shows schematics of the setup and a double-clad fibre sensor with a pigmented dome.

In one experiment, sensors were suspended in air at room temperature and ambient pressure, and sensor signals were recorded with a sampling rate of 1 kHz, while the pulsed laser illuminated the dome. In a second experiment, the sensors were placed into a beaker of still water at room temperature, and the above measurements were repeated. In a third experiment, the measurements in water were repeated while the water was stirred by a magnetic stirrer on a low-speed setting.

### 4.2. Experimental Results

**Figure 3:**
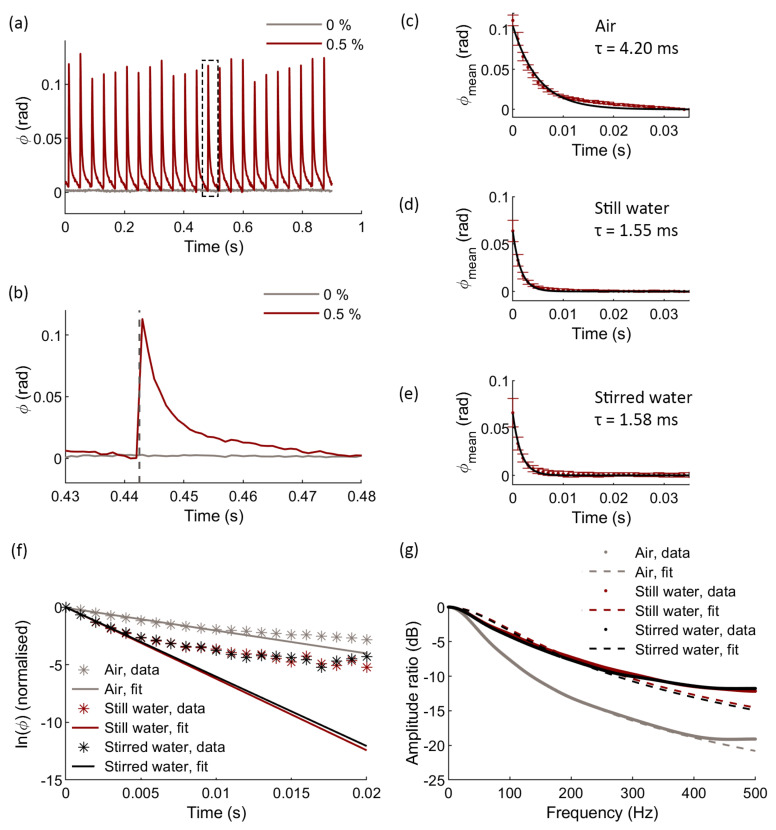
(**a**) Temperature sensor signals during illumination with 10 ns pulses of 532 nm laser light, repeating at 25 Hz; sensors were suspended in air; grey line: polydimethylsiloxane (PDMS) dome with 0% pigment; red line: PDMS dome with 0.5% pigment. (**b**) Enlarged view of one heating pulse; the dotted box in plot (**a**) indicates the region of data shown in plot (**b**). The dashed vertical line in plot (**b**) indicates the assumed time of one of the laser pulses, which occurs between 0.442 and 0.443 s. (**c**–**e**) Mean impulse response curves (N=22 to 24) induced by pulsed laser heating, obtained from the 0.5% pigment-containing dome sensor in different surrounding media: (**c**) air; (**d**) still water; (**e**) stirred water. The solid black lines are fitted curves of the form of Equation (9), from which the time constants τ were obtained. (**f**) Natural logarithm of data and fitted curves shown in in plots (**c**–**e**) versus time; (**g**) Sensor frequency response in air, still water and stirred water; the amplitude ratio was normalised by dividing by the maximum amplitude ratio occurring at 0 Hz; the dashed lines are fitted curves of the form $AR_{norm}\;=\;1/\surd \left(1\;+\;\omega^{2}\tau^{2}\right)$, where $AR_{norm}$ is the first-order instrument amplitude ratio given by Equation (10), normalised by dividing by the maximum amplitude ratio at ω=0 rad/s.

Figure 3a shows signals obtained from two of the sensors while suspended in air. The first sensor had a dome formed from pure PDMS (with no added pigment); the second sensor had a PDMS dome containing 0.5% (*w*/*w*) red pigment. When exposed to the 532 nm pulsed laser light, the signal from the blank PDMS sensor remained steady; in the signal from the pigment-containing sensor, abrupt increases in the signal, followed by slower, exponential-like decays, were observed. This indicates that impulse-like temperature changes were generated in the dome by absorption of the laser light by the pigment. The sensor responses also showed good repeatability, with the impulse response curves having a consistent form and magnitude across multiple laser pulses.

Data were processed using Matlab R2020a. Each set of measurements comprised 900 samples, corresponding to a time period of 0.9 s, in which 22 to 24 laser pulses were delivered. The peaks in the sensor signals caused by the laser pulses were located and the subsequent 34 samples were selected and translated along the time axis such that each peak maximum occurred at t=0 s. The samples at each time point after the pulse were then averaged to obtain a mean sensor response curve for each data set. An exponential function with the form of Equation (9) was fitted to each mean response curve, and the fitted parameter τ was taken as the time constant for the sensor in that medium.

Figure 3c–e show the mean sensor response curves derived from one sensor in three different media, i.e., air, still water and stirred water. The time constant was 4.20 ± 0.30 ms in air, 1.55 ± 0.07 ms in still water, and 1.58 ± 0.06 ms in stirred water, where the uncertainties represent the 95% confidence intervals in the fitted parameter τ. Time constants were determined experimentally for eight other pigment-containing sensors, in air and still water, and were found to be in the range 2.55 ± 0.28 ms to 6.24 ± 0.31 ms in air, and 1.24 ± 0.08 ms to 2.37 ± 0.24 ms in still water. Four of the sensors were tested in stirred water, and the time constants were in the range 1.20 ± 0.06 ms to 1.83 ± 0.09 ms. The sensor responses to optical heating by the laser pulses were highly consistent and repeatable, both within a single sensor and between different sensors. The time constants were generally larger for thicker domes, although the time constant and dome length were only weakly correlated for the measurements taken in still water (R^2^ = 0.45, compared to R^2^ = 0.72 for air and R^2^ = 0.95 for stirred water).

Upon comparing the data with the fitted curves in Figure 3c–f, deviations of the data from Equation (9) are apparent after a time of approximately 10 ms for air, and 4 ms for both still and stirred water. The fitting results suggest that some of the assumptions used to derive Equations (5)–(10) are invalid. In particular, we assumed homogeneous temperatures in the dome and the surrounding medium, and that heat transfer occurs only between the dome and the medium (with no heat transfer via the optical fibre). These assumptions will be relaxed in Section 5, where we investigate the sensors’ dynamic response using a finite element (FE) computational model.

The response curves of the sensors to the impulse-like optical heating were also used to estimate the sensors’ frequency responses. The peaks in each set of data were located, and the 35 samples centred on the peak were selected and translated along the time axis so that each set of samples began at t=0 s. The samples at each time point were then averaged to obtain a mean signal. After subtracting the baseline, zero-padding and windowing, the mean signal was Fourier transformed, and the resulting normalised frequency spectra are shown for one of the sensors in Figure 3g. For this sensor, the –6 dB bandwidth was 78 Hz in air, 158 Hz in still water, and 148 Hz in stirred water. The –6 dB bandwidths for the other sensors were in the range 66 Hz to 94 Hz in air, 42 Hz to 178 Hz in still water and 132 Hz to 183 Hz in stirred water. The data were fitted to curves of the form of the amplitude ratio of Equation (10). Again, the best fit is obtained for the measurements in air (R^2^ > 0.99), with greater deviations from the first order model observed in both still and stirred water (R^2^ = 0.97 and 0.95, respectively).

## 5. Dynamic Characterisation: Computational Modelling

The above experiment demonstrated the sensors’ response to a sudden impulse of heat generated within the dome itself. However, under normal operational conditions, the sensor will be responding to temperature changes in the surrounding medium. Creating well-known and reproducible dynamic temperature changes in the medium is challenging to perform experimentally: the plunge method may be used to produce a temperature step [17,18,35,36,50], and a ramp input may be produced by rapid insertion into an oven [37,68,70]; however, there are limitations to these methods as described in Section 1. Therefore, a FE transient heat-transfer model was used to study the sensors’ response to changes in the temperature of the surrounding medium, and the experimental results described above were used to validate the model.

### 5.1. Simulation Setup

**Figure 4:**
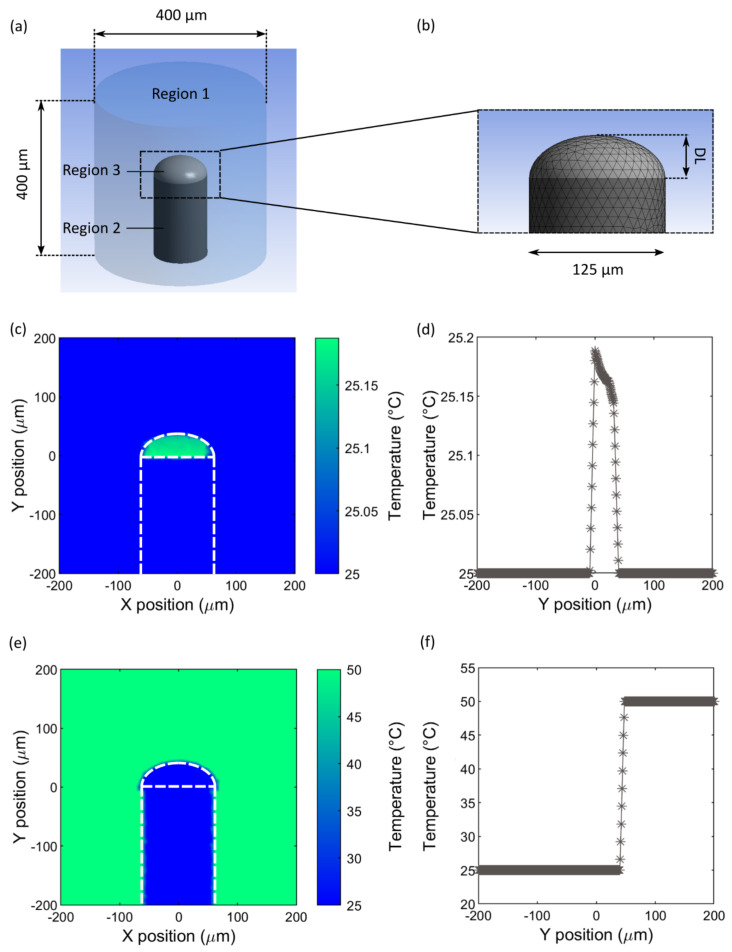
(**a**) Finite element (FE) model geometry; Region 1: surrounding medium (water), Region 2: optical fibre (fused silica); Region 3: PDMS dome. (**b**) Enlarged section of the finite element mesh (surrounding medium not shown), in the area indicated by the dotted box in (**a**); DL: dome length. (**c**) Initial temperature distribution for the FE model in the *x-y* plane at z=0, for the optical heating case; DL = 32 μm. (**d**) Initial temperature versus *y*-axis position for the optical heating case. (**e**) Initial temperature distribution for the FE model in the *x-y* plane at z=0, for the temperature step case; DL = 40 μm. In (**c**,**e**), the white dashed lines indicate the boundaries of the fibre and dome regions. (**f**) Initial temperature distribution versus *y*-axis position for the temperature step case.

The model was developed using Ansys Mechanical 19.0. The model geometry includes three subdomains denoted Region 1, Region 2 and Region 3, representing the surrounding medium (water), the fibre (fused silica) and the polymer dome (PDMS), respectively, as shown in Figure 4a,b.

Each region was assigned thermal properties required for computation of heat transfer, i.e., thermal conductivity, specific heat capacity and density, using values from the literature. The values used in the model, and their sources, are shown in Table 1. All properties were assumed to be isotropic, homogeneous and temperature-independent for the purposes of this study.

#### 5.1.1. Boundary Conditions

Fixed temperature boundary conditions were specified at the side and top surfaces bounding the computational domain in the model, i.e., it was assumed that far from the sensor element, the surrounding medium is maintained at a constant temperature. This is consistent with the conditions in the experiments, as the sensors were either suspended in air, or immersed in a beaker of water that was large compared to the expected size of the heated region of the surrounding medium. This is also expected to be the case when these devices are used in-vivo, due to temperature regulation inside the body. The specific fixed temperature depended on the initial temperature conditions (see next section): for the optical heating case, it was set to 25 °C, and for the temperature step case, it was set to 50 °C. The surface forming the lower boundary of the computational domain, which contained the fibre cross-section, was specified as having zero temperature gradient across this surface. This is a reasonable assumption if the edge of the computational domain is far enough from the dome structure that the temperature distribution could be assumed to be uniform in the y-direction. It was expected that this would be the case in our sensors, due to the distance of the lower boundary from the heated region, and the uniform cross-section of the structure in the y-direction in this area. We also optimised the domain size to ensure that the domain boundaries did not influence the results (see Section 5.1.4).

#### 5.1.2. Initial Temperature Distribution: Optical Heating

In the first instance, the model was given an initial temperature distribution based on optical heating of the dome caused by a laser pulse, to allow for comparison with the experimental results. The temperature distribution was calculated using the following equation, which was derived from the Beer–Lambert Law:

(11) $$\Delta T=\frac{I_{0}dt\mu_{a}}{C\rho \Omega r^{2}}\exp\left(-\;\mu_{a}r\right)$$

where ΔT is the increase in dome temperature above ambient temperature (25 °C), $I_{0}$ is the peak pulse power of the 532 nm laser, dt is the laser pulse duration, $\mu_{a}$ is the absorption coefficient of the dome at 532 nm, C is the specific heat capacity of the dome, ρ is the density of the dome, Ω is the solid angle of the incident beam emerging from the fibre, and r is the radial distance from the fibre facet.

The peak power of the laser was estimated by measuring the average power at 532 nm emerging from a bare 90° cleaved double-clad fibre (DCF13, Thorlabs, Newton, NJ, USA), and dividing by the duty cycle; the average power measured at 1 kHz was 2.5 mW, giving a peak power of 250 W. We assumed that the peak power at 25 Hz was similar, because the average power at 25 Hz was too low to measure directly with the available equipment.

The absorption coefficients of the pigment-containing domes were estimated using the concentration of pigment included in the dome material, and the relationship of absorption coefficient to concentration of the pigment discussed in Section 4. We assumed that there was no optical scattering in the dome.

The solid angle of the beam was estimated using the numerical aperture of 0.2 for the first cladding of the double-clad fibres, as specified by the manufacturer. The radius r was measured from the origin of the cone formed by the rays emerging from the fibre facet at the maximum acceptance angle. We assumed no angular dependence of the incident beam intensity. Finally, we used the specific heat capacity and density for PDMS shown in Table 1.

The initial temperature distribution is shown in Figure 4c,d. Figure 4c shows a temperature map in the *x-y* plane, and the temperature is plotted as a function of position along the *y*-axis at (*x*, *z*) = 0, in Figure 4d. For this simulation, a dome length of 32 µm and an absorption coefficient of 6.4 mm^−1^ were chosen to correspond to the characteristics of the sensor that produced the results in Figure 3. According to this model, the maximum temperature rise induced by the laser pulse is approximately 0.18 °C.

#### 5.1.3. Initial Temperature Distribution: Step Input

In subsequent simulations, the model was used to investigate the sensor’s response to temperature changes in the surrounding medium. A temperature step input was chosen to allow comparison with the first-order instrument model of Section 2. The dome and fibre were initially at a uniform temperature of 25 °C, and the surrounding medium was at a uniform temperature of 50 °C, as shown in Figure 4e,f.

#### 5.1.4. Mesh and Domain Optimisation

To optimise the balance between accuracy and time of computations, the length and diameter of the computational domain were set to 400 µm, and the maximum edge length of the mesh elements was set to 10 µm. A simulation was also performed with a computational domain size of 800 µm, and a further simulation was performed using a domain size of 400 µm and a mesh element maximum edge length of 8 µm. In both cases, it was found that the fitted parameter τ fell within the 95% confidence interval of the value of τ obtained with the original computational domain and mesh size.

#### 5.1.5. Solution

The transient heat transfer equations were solved using the Ansys Mechanical Heat Transfer module, with the assumption of a still surrounding medium, i.e., no flows or currents in the water. The solution was obtained using automatic time stepping for between 300 and 310 time steps, with a median time step size of 50 µs (the exact number and spacing of the time steps was automatically determined by the solver and was slightly different for each run). A final time point of 0.375 s was determined to ensure that the dome and fibre had reached thermal equilibrium with the water by the end of the simulation. For the temperature step model, the simulation was repeated with five different dome lengths: 30 µm, 35 µm, 40 µm, 45 µm and 50 μm. All simulated data were exported for further processing using Matlab R2020a.

### 5.2. Simulation Results

#### 5.2.1. Optical Heating Results and Comparison with Experiment

**Figure 5:**
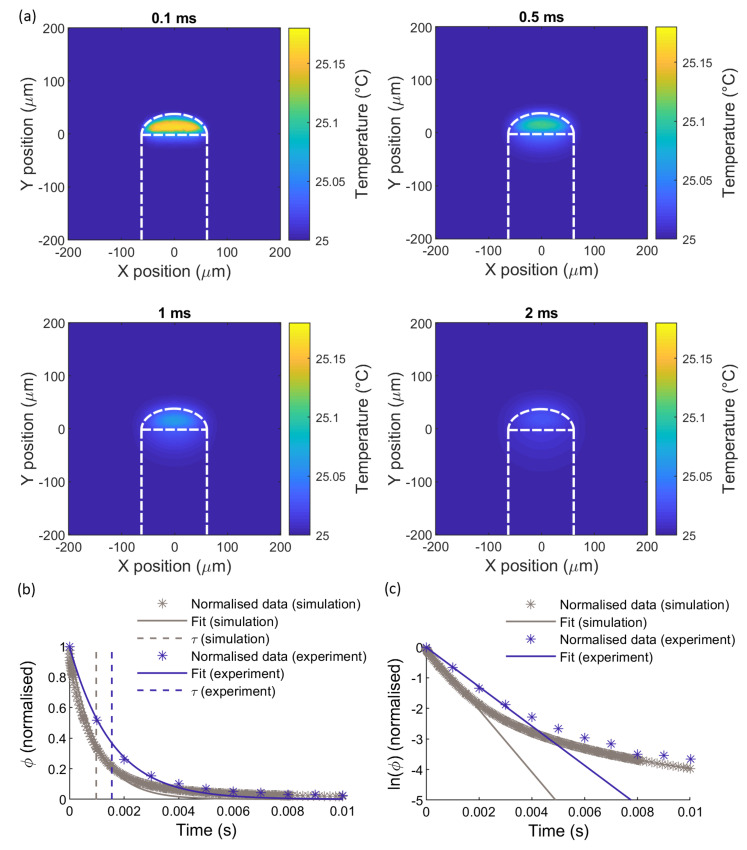
Laser-heated dome sensor simulation results: (**a**): temperature distribution in the x-y plane at different time points; (**b**) expected sensor signal calculated using the simulated temperature distributions, compared with experimental data. Both simulated and experimental data were normalised by subtracting the baseline and dividing by $\phi \left(t=0\right)$. The solid lines are fitted impulse response curves of the form of Equation (9), and the dashed lines indicate the time constants obtained from the fitted coefficient τ. Only data for t ≤ 0.01 s are shown, to show the differences in the two curves more clearly. (**c**) Natural logarithm of the data and fitted curves versus time for simulation and experiment.

Figure 5a shows simulated temperature distributions in the *x*-*y* plane (*z* = 0) at four different time points, for a 32 μm thick pigmented dome subjected to a temperature impulse produced by optical heating.

The temperature distribution at each time point in the simulation was interpolated on to a set of coordinates running along the optical (*y*) axis [(*x*, *z*) = 0], from the fibre facet to the edge of the dome, in increments of 0.8 µm. The interpolated temperatures at each *y*-position, $T_{i}$, were then used in Equation (4) to calculate the change in sensor signal, $\phi_{i},$ at each y position, and the sum of $\phi_{i}$ was calculated to obtain the sensor signal ϕ at that time point. Values used for the calculation were: α = 340 × 10^−6^ °C^−1^ [65]; $n_{0}$ = 1.3997 [65]; $\lambda_{c}$ = 1550 nm; for the thermo-optic coefficient of PDMS, we adopted dn/dT = −1 × 10^−4^ °C^−1^_,_ following Dong [81]; although lower values have been reported [82,83], these gave predicted temperature sensitivities according to Equation (4) that were much lower than those observed experimentally. The expected sensor signal, based on this calculation, is shown as a function of time in Figure 5b, where the simulated sensor signal is compared with the experimental results from Section 4.

In Figure 5b, the simulated and experimental data are normalised by dividing ϕ by $\phi \left(t=0\right)$ to facilitate comparison. In the experiment, the initial increase in ϕ observed after the laser pulse was 0.064 rad, which is several times larger than the increase predicted by the model (0.016 rad). The time constants obtained from the simulated and experimental data are 0.98 ms and 1.6 ms, respectively. The source of these discrepancies between the simulated and experimental data is unclear, but it could be due to uncertainties in our estimates of the various thermal, optical and geometric parameters used in the model.

The simulated and experimental data agree well in terms of the form of the impulse response and the order of magnitude of the time constant. Figure 5c shows the natural logarithm of the data and fitted curves, which emphasises the deviation of the data from the fit. The simulated data begin to deviate from the fitted curve at approximately 1.5 ms, confirming the departure from first-order instrument behaviour that was observed in the experiments.

#### 5.2.2. Temperature Step Input Results

**Figure 6:**
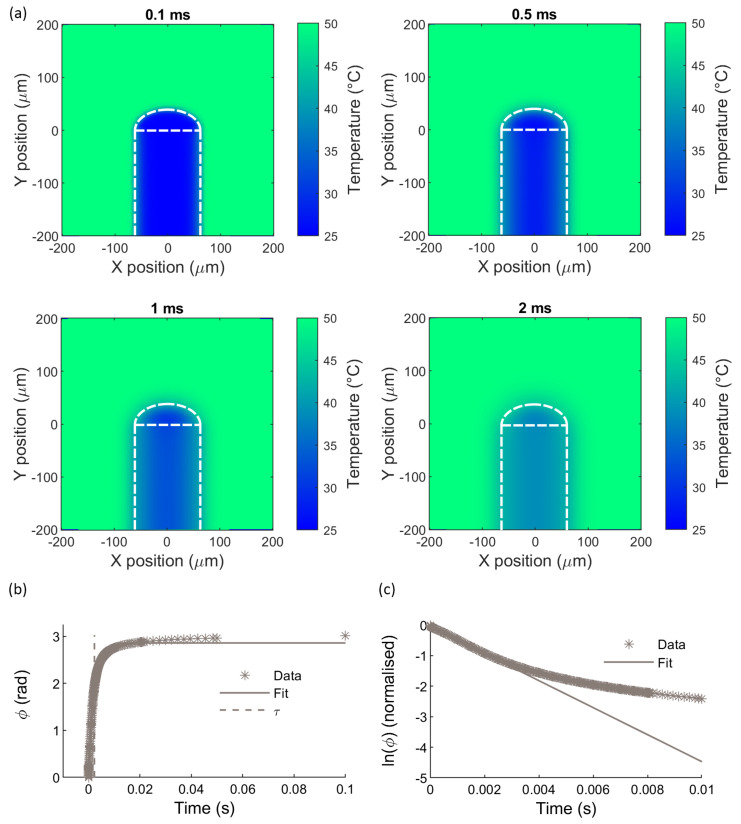
(**a**) Temperature maps in the *x*-*y* plane at *z* = 0, at different time points in the temperature step simulation. The surrounding medium had an initial temperature of 50 °C, and the dome and fibre were at an initial temperature of 25 °C. (**b**) Sensor signal versus time, in response to the step temperature change, as obtained from the simulated temperature distributions. The solid line indicates the exponential fit, and the dashed vertical line indicates the time constant obtained from the fit. Only data for t ≤ 0.1 s are shown, to show the initial rapid increase in the sensor signal more clearly. (**c**) Plot comparing the natural logarithm of the simulated sensor signal and fit, for t ≤ 0.01 s.

Figure 6 shows simulated temperature distributions in the *x*-*y* plane (*z* = 0) for a 40 um dome subjected to a temperature step in the surrounding medium, at four different time points in the simulation. The procedure described in Section 5.2.1 was used to convert the simulated temperature distributions into the expected sensor response to the step temperature change.

The sensor signal ϕ as a function of time is plotted in Figure 6b, along with a fitted curve with the form of Equation (8). The time constant obtained from the fit is 2.26 ± 0.06 ms. The time constants obtained for other dome lengths were 2.00 ± 0.06 ms, 2.11 ± 0.06 ms, 2.39 ± 0.06 ms and 2.52 ± 0.05 ms, for domes with thicknesses of 30 μm, 35 μm, 45 μm and 50 μm, respectively, where the uncertainties represent the 95% confidence intervals in the fitted parameter τ.

As with the experimental data and previous simulation results, the simulated data and fitted curve deviate from one another, particularly after 0.1 s. These deviations are emphasised in Figure 6c, where the natural logarithm of the normalised simulated data and fitted curves are plotted. To investigate the causes of the discrepancy, several additional simulations were run; these included modelling the fibre and dome only, with no surrounding medium and the temperature held constant at the outer boundaries of the dome and fibre; modelling the dome surrounded by the medium with no fibre present; and modelling a dome sensor with very high thermal conductivity (1000 Wm^−1^K^−1^). The resulting simulated sensor signals for each model are shown in Appendix A. The model where no medium was present yielded the closest fit to the first-order step response equation, with an R^2^ value of greater than 0.99, as compared to 0.99 for the original model, 0.99 for the model with no fibre, and 0.90 for the model with a highly conducting dome. Taken together, these results suggest that deviation from first-order behaviour is primarily due to an inhomogeneous temperature field developing in the medium surrounding the sensor.

## 6. Discussion

We have investigated the dynamic response of a fibre-optic temperature sensor that was developed for integration into minimally invasive surgical devices. We used an experimental method employing direct optical heating with a pulsed laser, to produce an impulse-like temperature profile in the dome, and a FE transient heat-transfer model to examine the sensors’ response to a temperature step in the surrounding medium. A temperature impulse was also simulated to validate the model against the experimental results. Both the experiments and the simulations showed that the time constants of the sensors were on the order of milliseconds.

Static calibration showed that the mean limit of detection of the sensors was 0.077 °C. All three sensors used for the static calibrations showed similar performance: a linear relationship was found between sensor signal and temperature, and the temperature sensitivities were similar, indicating good reproducibility. Polymers with larger thermal expansion coefficients or less negative thermo-optic coefficients could be used to enhance sensitivity and, therefore, lower the detection limit, and reflective coatings or materials with a stronger refractive index contrast compared to the fibre index could be used to increase the interference fringe visibility and, therefore, the signal to noise ratio (SNR) of the interferometric signal. However, higher thermal sensitivity in polymer-based FP fibre-optic sensors has been linked to higher loss modulus of the polymer material used [84], which could impact the dynamic characteristics of the sensor response, so there is likely to be a trade-off when selecting suitable materials.

The experiments found that the time constants of the sensors were in the range 2.55 ms to 6.24 ms in air, 1.24 ms to 2.37 ms in still water and 1.20 ms to 1.83 ms in stirred water. These results compare favourably with response times measured in FBG based sensors [17,18,35,36], and to electrical temperature sensors that have been characterized using optical heating methods [71,73]; faster response times have been measured with fibre optic temperature sensors based on silicon FP cavities [50] and thin-film thermocouples [72]. The sensor responses to optical heating were also highly repeatable within each sensor, showing impulse response curves having a consistent form and magnitude across multiple laser pulses, and reproducible, with similar measured impulse response curves and time constants across all sensors tested.

There was a weak correlation between time constant and dome thickness (R^2^ = 0.45) for still water, with stronger correlations in air (R^2^ = 0.72) and stirred water (R^2^ = 0.95). The correlation was expected to be strong, since a thicker dome implies a larger dome volume and, therefore, a longer delay in heat transmission to the central region of the dome. The weaker-than-expected correlation in still water could be due to disturbance of the temperature distribution in the water around the sensor after insertion; without stirring, a heterogeneous temperature field may have developed around the probe, giving rise to larger uncertainties in the measured time constants. The difference in time constants in air and water demonstrates the importance of characterising the sensors in the surrounding medium in which they will ultimately be used. However, the experimental time constants were similar in both still and stirred water, suggesting that conduction was a more prevalent heat transfer mechanism than convection under the experimental conditions used.

The frequency response of the sensors was also estimated by Fourier-transformation of the impulse response produced by optical heating, and the –6 dB bandwidths were in the range 66 Hz to 94 Hz in air, 42 Hz to 178 Hz in still water, and 132 Hz to 183 Hz in stirred water. These sensors, therefore, have the potential to detect thermal tagging and photothermal signals at a wide range of modulation rates [25], to resolve intra-coronary temperatures with spatial and blood pressure dependencies [85,86] and to measure rapidly changing temperatures with high temporal resolution for thermal treatment monitoring [19].

The FE model provided good agreement with the experimental results for the case of a temperature impulse produced by optical heating. Although there was some discrepancy in the initial temperature rise and the time constant, this was likely to be a result of uncertainties in the geometric, optical and thermal parameters used in the model; both sets of results showed the same exponential-like impulse response curve, with a time constant on the order of 1 ms to 2 ms. This agreement validated the model for the subsequent investigation of the sensor response to a temperature step.

We used the model to investigate the responses of the sensors to a temperature step input, for a range of dome lengths. These simulations confirmed the millisecond-order response times of the sensors, with time constants ranging from 2.00 ms for a 35 μm dome to 2.52 ms for a 50 μm dome. The simulated time constants followed the same trend as the experimental data, where the time constant increased with increasing dome length, but the simulated time constants were mostly larger than the experimental values for still water. This can be explained by the fact that in the experiment, the sensor element was heated directly, and heat was then transferred away through all surfaces of the dome, including via the dome–fibre interface. In the temperature step simulation, the temperature of the surrounding water changed, and heat was transferred into the dome only through the surface of the dome in contact with the water. Heat transfer to the dome through the dome–fibre interface could only take place after conduction through the fibre, thereby increasing the time constants of the sensors in the simulation. Since the sensors will be heated by the surrounding medium under normal operating conditions, the simulated time constants are likely to be closer to the true time constants of the sensors during typical usage.

Both the experimental and simulated results deviated from the simple analytical model presented in Section 2, which assumed first-order instrument behaviour. The simulations suggest that the greatest contributor to this deviation is the development of an inhomogeneous temperature field in the water surrounding the sensor. This justifies the need for FE simulations, as the sensor’s dynamic response cannot be fully understood with the analytical model presented here. It also underlines the importance of characterising the dynamic response of the sensor in the medium in which the sensor is to be used, since the sensor and region of fluid around it act as a combined system.

The FE model we have developed could be used to optimise sensor designs for greater sensitivity or faster response, depending on the requirements of the application; investigate the perturbing effect of the sensors on thermal distributions in the surrounding medium [16]; and evaluate the effects of sensor packaging [36]. Sensor optimisation could include increasing the thermal diffusivity of the dome material to increase the speed of response, matching the thermal properties of the sensor with that of the surrounding medium, and enhancing the sensitivity with different dome materials and geometries.

The FE model has the several limitations: the thermal properties of the sensor materials were not accurately known, and the model used values from literature. The model was based on conduction only, neglecting heat transfer via convection; in most surgical applications, significant fluid flows are likely to be present (e.g., blood flow in arteries), so an improved model would include fluid movement around the sensor. It was assumed that, far from the dome, there were no temperature gradients along the fibre axis; in a minimally invasive procedure, the sensor element would be placed inside the body while the connectorised end would be external to the body; therefore, temperature gradients along the fibre could be significant, depending on the depth of the sensor inside the body. Finally, the FE model does not include physical changes in dimensions of the dome arising from thermal expansion (<1 μm for the temperature ranges considered here).

## 7. Conclusions

We have developed a novel experimental paradigm and a finite element transient heat transfer model to investigate the dynamic behaviour of fibre-optic temperature sensors with polymer sensing elements. The sensors had time constants on the order of milliseconds, and –6 dB bandwidths of up to 178 Hz. The dynamic responses were strongly influenced by the surrounding medium, with measured time constants differing considerably in air and in water. The dynamic responses also deviated from first-order instrument behaviour, with simulations suggesting that the main cause of this was the influence of an inhomogeneous temperature distribution in the medium. Our results indicate that these sensors are well suited for many minimally invasive applications where measurement of rapidly varying temperatures is required.

## Figures and Tables

**Table 1:** Thermal property values used in the finite element (FE) model.

Property	Region 1: Water	Region 2: Fused Silica	Region 3: PDMS
Thermal conductivity (Wm^−1^ °C^−1^)	0.606523 [66]	1.37 [67]	0.27 [65]
Density (kgm^−3^)	997.05 [66]	2200 [67]	970 [80]
Specific heat capacity (Jkg^−1^ °C^−1^)	4181.3 [66]	745.1 [66]	1460 [80]

**Figure A1:**
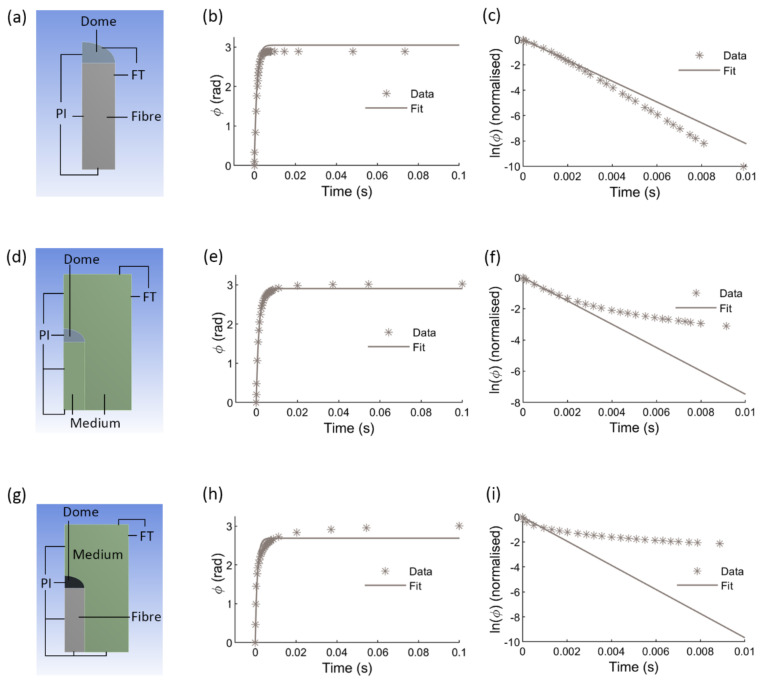
Results of additional temperature step simulations used to investigate the causes of the discrepancy between the simulated sensor data and first order instrument behaviour. These simulations were two-dimensional and axisymmetric, to reduce computation time; in all three models, perfectly insulating boundary conditions (denoted PI) were applied to the edge of the computational domain lying along the axis of symmetry and the lower edge of the computational domain; fixed temperature boundary conditions (denoted FT) were applied to the other edges of the computational domain. (**a**) Geometry for model of fibre and dome with no surrounding medium; (**b**) simulated sensor signal versus time, and fitted curve of the form of Equation (8), for model of fibre and dome with no medium; (**c**) natural logarithm of simulated data and fit shown in (**b**), versus time; (**d**) geometry for model of dome surrounded by medium with no fibre present; (**e**) simulated sensor signal and fitted curve for model with no fibre present; (**f**) natural logarithm of data and fitted curve shown in (**e**) versus time; (**g**) geometry for model with high thermal conductivity of the dome (1000 Wm^−1^K^−1^); (**h**) simulated sensor signal and fitted curve for model with the highly thermally conducting dome; (**i**) natural logarithm of the simulated data and fitted curve shown in (**h**), versus time.

## Data Availability

Data is contained within the supplementary material.

## Supplementary Materials

The following are available online at https://www.mdpi.com/1424-8220/21/1/221/s1: raw data for the results presented in this study.
